# Estimation of Urine Flow Velocity Using Millimeter-Wave FMCW Radar

**DOI:** 10.3390/s22239402

**Published:** 2022-12-02

**Authors:** Yingnan Qi, Hyounjoong Kong, Youngwook Kim

**Affiliations:** 1 Department of Electrical and Computer Engineering, California State University, Fresno, CA 93740, USA; 2 Transdisciplinary Department of Medicine & Advanced Technology, Seoul National University, Seoul 03080, Republic of Korea; 3 Department of Electronic Engineering, Sogang University, Seoul 04107, Republic of Korea

**Keywords:** FMCW radar, range-Doppler diagram, urine rate measurements, uroflowmetry, remote health care

## Abstract

This study investigated the feasibility of remotely estimating the urinary flow velocity of a human subject with high accuracy using millimeter-wave radar. Uroflowmetry is a measurement that involves the speed and volume of voided urine to diagnose benign prostatic hyperplasia or bladder abnormalities. Traditionally, the urine velocity during urination has been determined indirectly by analyzing the urine weight during urination. The maximum velocity and urination pattern were then used as a reference to determine the health condition of the prostate and bladder. The traditional uroflowmetry comprises an indirect measurement related to the flow path to the reservoir that causes time delay and water waves that impact the weight. We proposed radar-based uroflowmetry to directly measure the velocity of urine flow, which is more accurate. We exploited Frequency-Modulated Continuous-Wave (FMCW) radar that provides a range-Doppler diagram, allowing extraction of the velocity of a target at a certain range. To verify the proposed method, first, we measured water speed from a water hose using radar and compared it to a calculated value. Next, to emulate the urination scenario, we used a squeezable dummy bladder to create a streamlined water flow in front of the millimeter-wave FMCW radar. We validated the result by concurrently employing the traditional uroflowmetry that is based on a weight sensor to compare the results with the proposed radar-based method. The comparison of the two results confirmed that radar velocity estimation can yield results, confirmed by the traditional method, while demonstrating more detailed features of urination.

## 1. Introduction

The measurement of the urine flow rate during urination is critical in diagnosing benign prostatic hyperplasia (BPH), low urinary tract symptoms (LUTS), and bladder outlet obstruction (BOO). BPH is a medical condition involving enlargement of the prostate that can lead to obstruction of the urinary tract, leading to slow urination. LUTS is a condition that can lead to hesitancy or intermittent streams, which implies a reduction or even no velocity of urine flow. Similarly, BOO is connected to slow urine and no urine streams [1].

The significance of urinary problems is reflected in a variety of statistics; for example, 14 million men in the United States and approximately 30 million worldwide have demonstrated signs of BPH. Reports have shown LUTS affecting up to 60% of the overall population, impacting 62.8% of men and 59.6% of women [2]. These diseases can lead to urinary hesitancy, a weak stream, prolonged voiding, and more, all of which can negatively impact the quality of life. LUTS can cause an uncontrollable urinary pattern on both conscious and unconscious levels. Urine leakage can erode confidence in life, leading to constant concerns. Furthermore, due to the nature of the disease, patients may feel uncomfortable about consulting a medical professional to discuss their symptoms openly in a timely manner in order to seek out the earliest possible treatment. One common trait of BPH, LUTS, and BOO is an abnormal urinary pattern. This pattern has a direct relationship with the urine flow rate. Thus, it is necessary to use an accurate, easy-to-use, sanitary, direct, and continuous method to estimate the urine flow velocity to diagnose human urodynamic health.

One traditional method of measuring urine flow velocity utilizes load cell sensors to obtain the amount of weight increase with respect to time. The velocity information can be calculated by finding the derivative of the change of weight over time, given the area of cross-section of the urethra. The physical design of the urine collection apparatus involves a spiral collector funnel that directs voided fluid to a scale for weighing. The spiral design acts as a mechanical low-pass filter that can remove drastic changes in weight with time. The advantages of this method include the ability to keep track of the overall weight of the fluid, as well as the rate of change of weight using a simple weight sensor. However, the method also has drawbacks, including that the measurement of velocity is conducted indirectly, making it vulnerable to a loss of accuracy due to spillage during urination [3]. Other complications, such as a mechanical low-pass filter, can alter the instantaneous urine velocity through time delay; moreover, the water wave in the container can affect the weight of the urine saved. In general, significant fluctuation of urine flow rate is observed due to the water wave. In addition, for sanitary purposes, medical personnel must clean the uroflowmetry apparatus thoroughly after each measurement, which is an unpleasant task and delays the measurement time in a clinic setting.

Another viable approach uses ultrasound devices to scan the bladder. An ultrasonic device can produce a visualization of the overall status of the bladder. This method requires the bladder to be scanned before and after urination. Based on the estimated volume change and the time elapsed, the indirect averaged velocity is calculated. This method offers the advantage of gaining information about the percentile of urination. However, the method’s drawbacks include the need for specialized ultrasound equipment and personnel for the measurement attempt [4]. In addition, the estimated velocity represents an averaged and not an instantaneous value.

In addition, there have been strong demands for devices that measure urine velocity remotely and personally. It is reported that the low visiting percentage of urinary disorder patients might be attributed to the lack of a convenient and private measurement of voiding dysfunction [5]. In [6], a small-sized vibration sensor that transforms vibration signals into uroflow during urination was proposed. The device was designed to assist in in-home monitoring of voiding status of patients. In [7], an artificial intelligence model was used to analyze the vibration data and predict six common patterns of uroflowmetry to diagnose voiding dysfunction. Acoustic uroflowmetry that utilizes urination sound was also suggested to estimate urine velocity [8,9]. However, those are indirect methods of measuring urine velocity, which may contain large errors and miss the variation details of urine velocity.

In this study, we proposed a method to directly measure urine rate using a millimeter-wave frequency-modulated continuous-wave (FMCW) radar. Measurement via radar has been used in target detection, range estimation, velocity estimation, and direction of arrival analysis. The advantages of using radar include precise measurements in range-Doppler with non-light dependency and the through-object capability [10,11]. Measuring urination through radar allows directly monitoring urine velocity instantaneously. In particular, the range to the target will be constant for this application; thus, velocity information can be extracted for a specified distance. Therefore, it can eliminate an unideal effect from the funnel and container.

First, we validated the proposed method by measuring water velocity from a garden hose placed 2 m away from the FMCW radar. The radar module was placed opposite to the direction of fluid flow. By measuring the height of the hose and the distance that the water traveled until it hit the ground, the speed of the water could be calculated. The result could be compared to the value from the radar. After verifying that the radar could detect the fluid speed, a measurement scenario that emulated urination was created. The dummy bladder that holds water was located next to a human subject and squeezed. Unlike the previous attempt, the fluid flow in this case was not constant; thus, the speed varied. A scale for weighing was included in the measurement as the reference urine flow rate measurement.

The remaining sections of this paper are as follows: Section 2 describes the principle of signal processing for FMCW radar, Section 3 describes the validation of fluid velocity measurement using radar, Section 4 discusses velocity measurement of fluid from a dummy bladder, and Section 5 presents the conclusions and discussions on potentials and limitations of the proposed method.

## 2. Radar Signal Processing

**Figure 1:**
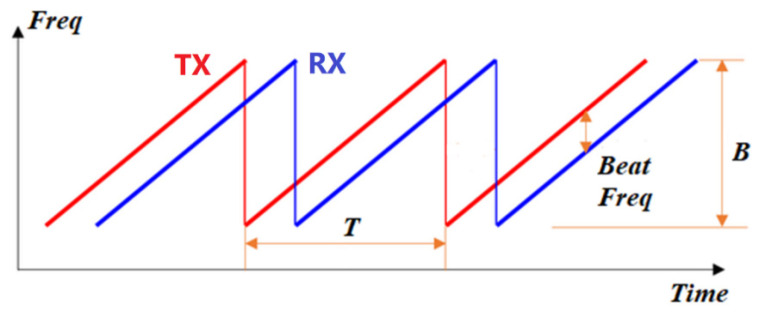
The frequency of FMCW radar with time (fast chirp signal). Tx is the transmitted signal, Rx is the received signal, *T* is the period, and *B* is the bandwidth.

Traditional CW radar has a major limitation: it cannot measure distance because it lacks a timing mark in the transmitting and receiving signals. Contrarily, FMCW radar has the capability of measuring the distance, as well as the velocity, of a target through modulating the transmitting frequency with time. Frequencies of the transmitting signal and the received signal are shown in Figure 1. Because it is simple to design and cost-effective, FMCW radar has been popular for short-range and mid-range applications. The use of fast chirp, of which the period ranges in micro-seconds, is advantageous in that it can provide information regarding target RCS distribution and micro-Doppler characteristics. Furthermore, it is straightforward to construct the range-Doppler diagram through the 2D-FFT. In the range-Doppler domain, it is convenient to detect a weak target because targets are separated by not only the range axis, but the Doppler axis.

All figures and tables should be cited in the main text as Figure 1, Table 1, etc.

The theoretical concept and the process of generating a 2D range-Doppler diagram using 2D FFT is described as follows [12]. The basic idea in FMCW is to generate a linear frequency ramp. The transmit frequency for one ramp with bandwidth *B* and duration *T* between [−*T*/2, *T*/2] can be expressed as:

(1) $$f_{T}\left(t\right)=f_{c}+\frac{B}{T}t$$

After integration, the phase of the transmitted signal of becomes:

(2) $$\phi_{T}\left(t\right)=2\pi \cdot \overset{t}{\underset{-T/2}{\int }}f_{T}\left(t\right)dt=2\pi \cdot \left(f_{c}t+\frac{1}{2}\cdot \frac{B}{T}t^{2}\right)-\phi_{To}$$

When τ is the time delay between transmitted signal and received signal, the phase of the down-converted signal $\Delta \phi \left(t\right)=\phi_{T}\left(t\right)-\phi_{T}\left(t-\tau \right)$ is:

(3) $$\Delta \phi \left(t\right)=2\pi \cdot \left(f_{c}\cdot \tau +\frac{B}{T}t\cdot \tau -\frac{B}{2T}\tau^{2}\right)$$

Because the time delay of τ is much smaller than *T*, the last term can be neglected. When a target is moving, the distance (*R*) should be modified including the velocity component (*v*) so that the time delay becomes τ=2(R+v⋅t)/c. This leads to:

(4) $$\Delta \phi \left(t\right)=2\pi \cdot \left[\frac{2f_{c}R}{c}+\left(\frac{2f_{c}v}{c}+\frac{2BR}{T\cdot c}\right)\cdot t+\frac{2B\cdot v}{T\cdot c}t^{2}\right]$$

The last term is called the range-Doppler coupling and can be ignored since it is a small value. Therefore, the generated frequency is:

(5) $$f_{IF}=\frac{2f_{c}v}{c}+\frac{2B\cdot R}{T\cdot c}$$

The received signal is sampled with an interval of *T_A_* (slow time), the samples are multiplied with a window function *w*(*n*), and a zero padding is performed. For the down conversion, we use a quadrature mixer so that the complex time domain signal can be constructed as:

(6) $$\;\;s_{B}\left(n\right)=A\cdot w\left(n\right)\cdot exp(j\cdot 2\pi \left[\frac{2f_{c}\cdot R}{c}+\left(\frac{2f_{c}\cdot v}{c}+\frac{2B\cdot R}{T\cdot c}\right)T_{A}n\right])$$

Here, we let *A* and *w*(*n*) as one for the simplification of the equation and assume the *L* frequency ramps. The ramp repetition interval is called $T_{RRI}$ (fast time). The resulting IF-signal is expressed as:

(7) $$s_{IF}\left(t\right)=\overset{L-1}{\underset{l=0}{\sum }}exp(j\cdot 2\pi \left[\frac{2f_{c}\cdot \left(R+v\cdot T_{RRI}l\right)}{c}+\left(\frac{2f_{c}\cdot v}{c}+\frac{2B\cdot \left(R+v\cdot T_{RRI}l\right)}{T\cdot c}\right)t\right])\cdot rect\left(\frac{t-l\cdot T_{RRI}}{T}\right)$$

$$\approx e^{j\cdot 4\pi \cdot fc\cdot R/c}\overset{L-1}{\underset{l=0}{\sum }}exp(j\cdot 2\pi \left[\frac{2f_{c}\cdot v\cdot T_{RRI}l}{c}+\left(\frac{2f_{c}\cdot v}{c}+\frac{2BR}{T\cdot c}\right)t\right])\cdot rect\left(\frac{t-l\cdot T_{RRI}}{T}\right)$$

If two-dimensional Fourier transform is performed:

(8) $$S_{2D}\left(k,p\right)=e^{j\cdot 4\pi \cdot f_{c}R/c}\overset{L-1}{\underset{l=0}{\sum }}\overset{N-1}{\underset{n=0}{\sum }}e^{j\cdot 4\pi \cdot \frac{vT_{RRI}f_{c}l}{c}}exp(j\cdot 2\pi \left[\frac{2f_{c}\cdot v}{c}+\frac{2B\cdot R}{T\cdot c})\cdot n\cdot T_{A}\right])\cdot exp-j2\pi \left(\frac{l\cdot p}{L_{Z}}+\frac{n\cdot k}{N_{Z}}\right)$$

where *Lz* and *Nz* are the matrix size after zero padding. Finally, $S_{2D}(k,p)$ is the representation of range-Doppler information. In this study, range-Doppler diagrams were used as the primary analytical technique because we can detect a target velocity at a certain range bin.

For measurement, we employed millimeter wave AWR1243 FMCW radar manufactured by TI as the main equipment in this study. Radar setup parameters are crucial for the detection of urine flow rate. In our setup, the bandwidth used was 4 GHz with a starting frequency at 77 GHz. The resulting range resolution is 3.75 cm. The chirp slope was 80 MHz/us. The sampling rate used was 2400 ksps. The designed chirp duration was 22 usec active and 5 usec idle. The main structure contains 100 ADC samples per chirp, and 100 chirps were used per frame. The total number of frames used was 2200 frames, with a frame periodicity of 11.2 ms. Data were captured through mmwave-Studio from TI, and we used Matlab for data processing.

The Doppler frequency is proportional to the carrier frequency. Therefore, a higher frequency is preferable to detect low-velocity targets with high accuracy. As the urine speed is around 1–5 m/s, we chose radar operating in the millimeter-wave range to obtain a higher Doppler frequency. The bandwidth of radar determines the range resolution, as well as the maximum range of detection given the ADC sampling rate. As this application is limited in a short range, we have increased the bandwidth as much as we can.

## 3. Validation of Fluid Velocity Measurement

**Figure 2:**
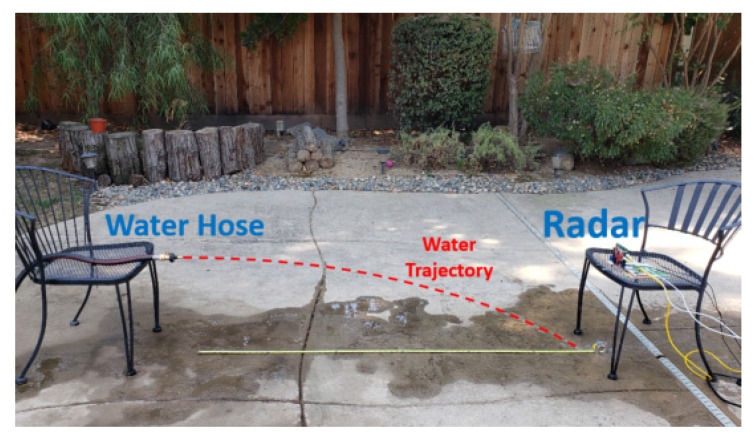
The water velocity measurement using a garden hose.

Doppler radar has been employed to measure the velocity on the water surface [13,14] to estimate the water flow in a creek or river. In [15], synthetic aperture radar (SAR) measured the surface water flow speed of a river. However, to the best of the authors’ knowledge, the velocity of water ejected from a tube has not been researched yet. To validate the accuracy of measuring the velocity of liquid flow ejected from a tube using radar, we designed and conducted an experiment using a water hose. In this experiment, a stream of water was produced by a garden hose with different water pressure to reach different distances on the ground. The distances ranged from 0.3 m to 1.8 m, as shown in Table 1. Because the accurate control of the water pressure is not easy, we measured the distance that the water falls on the ground. The elevation of the hose was set to 0.72 m, as shown in Figure 2.

The velocity of water flow can be calculated in two ways. The first method is to derive the velocity given the total volume of water emerged and the cross-sectional area of the fluid dispenser. However, this method is prone to error because the accurate estimation of the cross-section area is tricky. The second method is to calculate the velocity by modeling water as a particle subjected to free-fall projectile motion. We used the latter method because it did not require information about the cross-section area of the dispenser. When the height of the hose and the traveled distance were known, the speed of the water could be mathematically calculated as follows:

(9) $$v=D\;\cdot \;\sqrt{\frac{g}{2h}}$$

where *v* is the velocity of water, *D* is the distance the water travels, *g* is the gravity, and *h* is the height of the water hose.

**Figure 3:**
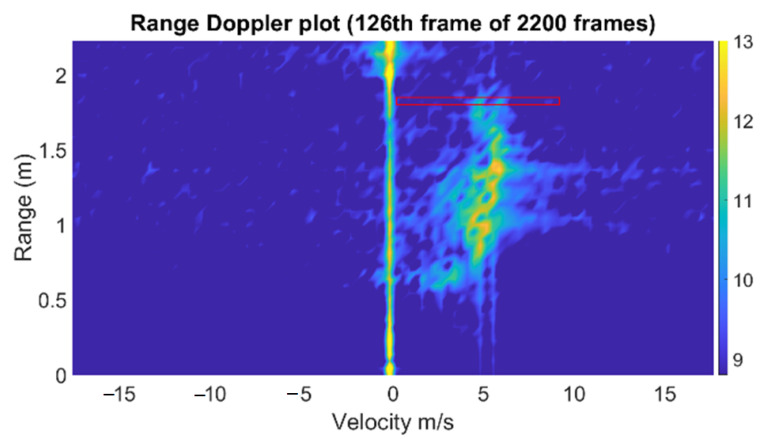
The range-Doppler diagram of water from a hose. The speed of water at 1.8 m (red box) was estimated.

After measurement, the range-Doppler diagram was generated with a 2D FFT technique. The importance of this plot is that it could provide velocity information depending on the range. Figure 3 shows the range-Doppler diagram of the water from the hose. The water continuously emerged from the hose with almost the same speed; thus, the range-Doppler diagram does not noticeably change with time. The velocity information observably varies depending on the range that the water traveled because the water drops due to the influence of gravity. The radial velocity that the radar perceives decreases as the range gets closer. The most significant value in this case is the velocity of the water as it emerges from the hose. Because the distance of the hose from the radar is 1.8 m, we can focus on that range. However, there will still be a variance in velocity because water is fluid. The speed of various water particles differs due to friction from the tube’s surfaces. We select the velocity value with the high energy intensity, which is the median value in general.

Table 1 displays the velocity measured by radar and the calculated velocity at different distances water traveled. The measurement and analysis outcomes are similar, and the root mean square error is 0.101 m/s.

## 4. Velocity Measurement of Fluid from Dummy Bladder

**Figure 4:**
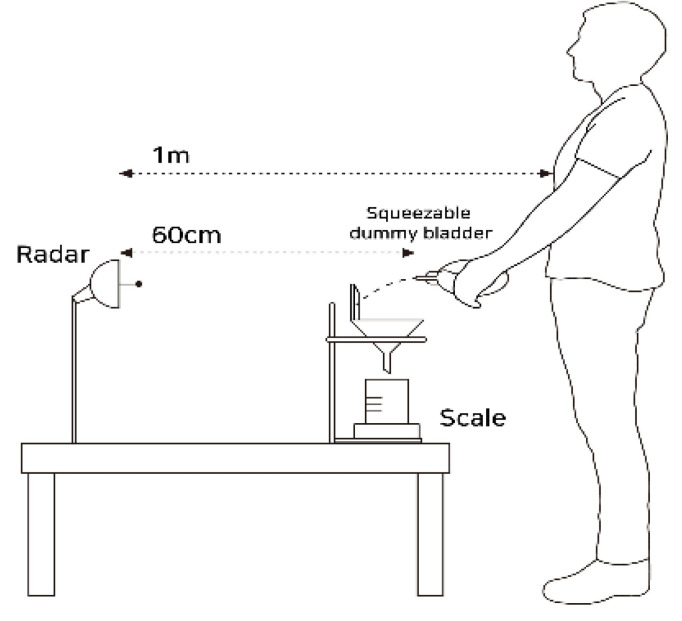
Lateral view of the experimental setup showing the position of the human target and the squeezable dummy bladder.

The next measurement setup was configured to emulate the urine voiding scenario. During the measurement, the human subject squeezed the dummy bladder, which is composed of a squeezable water container and water dispenser, to simulate the effect of urination. The bottle had a narrow tube and was located adjacent to the human body. The emitted water from the bottle was collected in a container. For the cross-validation, we included a means of traditional measurement by placing the fluid collector on top of the weighing scale and measuring the weight of the collector containing the water from the squeezed dummy bladder. The squeeze motion was limited to one gradual squeeze to simulate a urination scenario. At the same time, the millimeter-wave FMCW radar module was placed directly opposite the direction of fluid emission. The distance from the collector to the radar was 0.6 m, and the distance between the human target and the radar was 1 m, as shown in Figure 4. The measurement process took place concurrently with both the weight measurement and the radar measurement while the human subject squeezed the dummy bladder bottle. We have received institutional review board (IRB) approval from California State University, Fresno, for this measurement, under IRB-ECE-2021-01.

**Figure 5:**
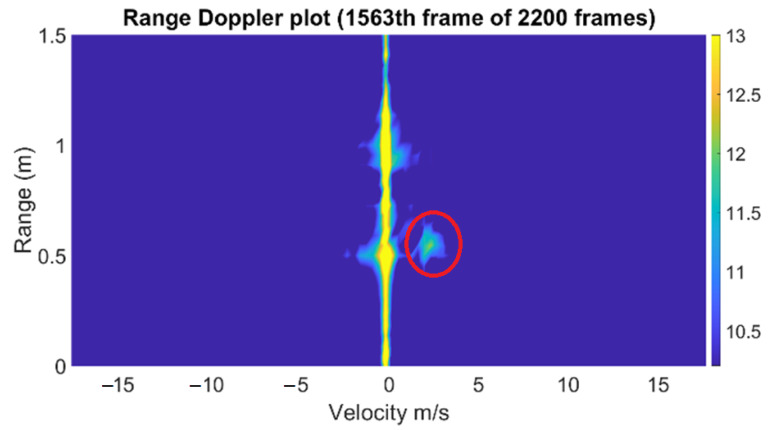
The range-Doppler diagram of water from a dummy bladder. The red circle shows the detected water from the dummy bladder.

In the same manner as the water hose measurement case, we extracted the velocity information from the range-Doppler diagram for each frame. Once again, for the given scenario, the range of the targets was fixed, and we only focused on the velocity at the range where the bottle was located. In Figure 5, the bright yellow spot indicates detected the water velocity of the dummy bladder in front of the radar. The figure presents the range-Doppler diagram depicted at one frame in which the indicated detected target was at 0.6 m distance, with an approximate velocity of 2.1 m/s.

In the range-Doppler diagram, we captured the velocity of the highest energy concentration. Compared to the previous measurement using a hose, it can be observed that the signal strength of the signature is much lower because the cross section of the tube of the dummy bladder is smaller than that of the water hose. In addition, the Doppler signature exists at only around 0.6 m, as the emitted water from the bottle was reflected from the wall of the fluid collector, as shown in Figure 4. For each frame, the same process of estimating the velocity in the range-Doppler diagram is performed, resulting in the time-varying water velocity graph. On the other hand, a spectrogram that shows the time-varying frequency is another method to observe the fluid velocity with time when a human squeezed the bottle. The short-time Fourier transform (STFT) can be applied to time series of the same range bin (slow time) to generate a spectrogram. However, the low resolution in the frequency domain, as well as the time domain, makes it not suitable to determine the sudden changes in Doppler frequency.

During the measurement process, the change of weight was recorded to find the reference flow rate. The traditional measurement of the urine flow rate was done by analyzing the rate of change of weight over time, which is a differentiation of weight. The velocity of water could be calculated using the following equation:

(10) $$v=\frac{weight\;Rate}{S}\;$$

where *S* is the area of the cross-section of the hose.

**Figure 6:**
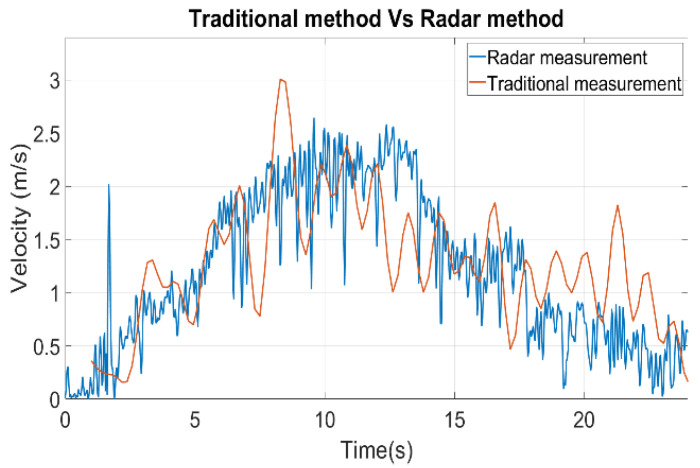
Trial 1, traditional measurement result plot vs. radar measurement result plot.

We conducted two trials to measure water velocity from squeezing a dummy bladder. The capacity of the dummy bladder was 200 mL. The resulting velocities using the weight measurement (red) and radar measurement (blue) are shown in Figure 6. As the figure shows, the traditional measurement using a scale and the radar measurement demonstrate a similar pattern. In contrast to the more noticeable fluctuation of the traditional method, the result from the radar is more solid, even though high-frequency components are observed. The low pass filter implemented with a finite impulse response eliminates the high-frequency components as a post-processing. The reason for fluctuation in the traditional method is because of the water wave in the container when the squeezed water is dropped to the container. The total weight of the water was 165.69 g from the weight scale, whereas the calculated weight from the radar is 167.42 g, assuming the density of water is 1 g/mL. The cross-section area S of the dummy bladder was estimated to be 6.6 mm^2^.

**Figure 7:**
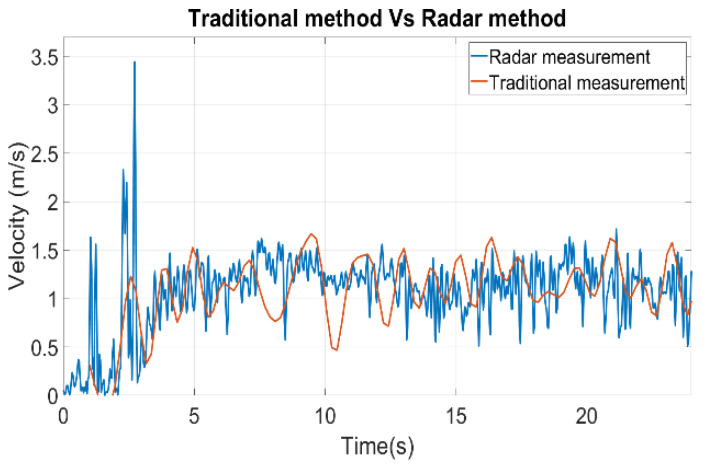
Trial 2, traditional measurement result plot vs. radar measurement result plot.

The results of the second trial are shown in Figure 7, which reveals that the radar measurement offered more accuracy and detailed information. Not only was the radar able to pick up a sudden change of velocity during the initial stage of the measurement around 2 s, but it also maintained its accuracy throughout the measurement compared to the traditional method. Once again, the traditional method demonstrated the fluctuation of the weight due to the water wave in the container. The total weight of the water was 165.37 g from the weight scale, whereas the calculated weight from the radar was 172.9 g. The error can be from the water due to spillage and velocity estimation error in the range-Doppler domain.

## 5. Conclusions

The measurement of urine flow rate using millimeter wave FMCW radar was proposed, and the concept was verified through measurement. Whereas current reference uroflowmetry is based on an indirect measurement that relies on the weight change resulting from urination over time, the proposed approach offers a direct method that can be more accurate. The performance of the traditional weight sensor-based uroflowmetry was affected by a mechanical delay of the water path and the water fluctuation of the container. In contrast, not only was the radar-based uroflowmetry able to measure the velocity of the fluid at the end of the hose where the water emerged, but the instantaneous velocity could be measured with precision. The measurement results from the squeezable dummy bladder bottle that mimicked urination showed that the radar could detect tiny streams of fluid, and the velocity components could be extracted from the range-Doppler domain. Conceivably, this radar-based method can provide a new standard for uroflowmetry. Furthermore, radar can be embedded inside a standard toilet that can log the urination pattern over a long time period, providing significant data to diagnose related diseases [16].

However, it should be noted that the proposed method will provide a voided urine volume with less accuracy compared to the traditional method, as the cross-section area of the urethra should be correctly assumed. In addition, the radar position needs to be carefully determined depending on the measurement setup as radar detects a radial velocity, especially when the direction of urine is not horizontal. Finally, this study provided only a proof of concept, so future clinical evaluation should be conducted to validate the proposed method from a clinical perspective.

## Figures and Tables

**Table 1:** Velocity comparison at different distances.

Distances (m)	Manual Calculation Using Equation (9) (m/s)	Radar Measurement (m/s)
0.36	0.939	1.07
0.61	1.59	1.66
0.96	2.50	2.61
1.15	3.00	2.85
1.49	3.88	3.92
1.78	4.64	4.70

## Data Availability

Not applicable.
